# Engaging young people in the design of a sexual reproductive health intervention: Lessons learnt from the Yathu Yathu (“For us, by us”) formative study in Zambia

**DOI:** 10.1186/s12913-021-06696-7

**Published:** 2021-07-29

**Authors:** Melvin Simuyaba, Bernadette Hensen, Mwelwa Phiri, Chisanga Mwansa, Lawrence Mwenge, Mutale Kabumbu, Steve Belemu, Kwame Shanaube, Ab Schaap, Sian Floyd, Sarah Fidler, Richard Hayes, Helen Ayles, Musonda Simwinga

**Affiliations:** 1 Zambart, Lusaka, Zambia; 2 Department of Clinical Research, Faculty of Infectious and Tropical Diseases, London School of Hygiene and Tropical Medicine, London, UK; 3 Department of Infectious Disease Epidemiology, Faculty of Epidemiology and Population Health, London School of Hygiene and Tropical Medicine, London, UK; 4 Department of Infectious Disease, Imperial College NIHR BRC, Imperial College, London, UK

**Keywords:** Sexual and reproductive health, Adolescents and young people, Qualitative, Engagement, Participatory, Incentivised, Community-based, Peer-led, Zambia

## Abstract

**Background:**

Meeting the sexual and reproductive health (SRH) needs of adolescents and young people (AYP) requires their meaningful engagement in intervention design. We describe an iterative process of engaging AYP to finalise the design of a community-based, peer-led and incentivised SRH intervention for AYP aged 15–24 in Lusaka and the lessons learnt.

**Methods:**

Between November 2018 and March 2019, 18 focus group discussions, eight in-depth interviews and six observations were conducted to assess AYP’s knowledge of HIV/SRH services, factors influencing AYP’s sexual behaviour and elicit views on core elements of a proposed intervention, including: community-based spaces (hubs) for service delivery, type of service providers and incentivising service use through prevention points cards (PPC; “loyalty” cards to gain points for accessing services and redeem these for rewards). A total of 230 AYP (15 participated twice in different research activities) and 21 adults (only participated in the community mapping discussions) participated in the research. Participants were purposively selected based on age, sex, where they lived and their roles in the study communities. Data were analysed thematically.

**Results:**

Alcohol and drug abuse, peer pressure, poverty, unemployment and limited recreation facilities influenced AYP’s sexual behaviours. Adolescent boys and young men lacked knowledge of contraceptive services and all AYP of pre and post exposure prophylaxis for HIV prevention. AYP stated a preference for accessing services at “hubs” located in the community rather than the health facility. AYP considered the age, sex and training of the providers when choosing whom they were comfortable accessing services from. PPCs were acceptable among AYP despite the loyalty card concept being new to them. AYP suggested financial and school support, electronic devices, clothing and food supplies as rewards.

**Conclusions:**

Engaging AYP in the design of an SRH intervention was feasible, informative and considered responsive to their needs. Although AYP’s suggestions were diverse, the iterative process of AYP engagement facilitated the design of an intervention that is informed by AYP and implementable.

**Trial registration:**

This formative study informed the design of this trial: ClinicalTrials.gov, NCT04060420. Registered 19 August, 2019.

## Background

Meeting the sexual and reproductive health (SRH) needs of adolescents and young people (AYP) aged 10–24 is a global public health challenge [1, 2]. AYP are at increased risk of contracting sexually transmitted infections (STIs), including HIV that can result in lifetime consequences [2–6]. Globally, HIV is one of the leading causes of death among AYP aged 10–19 [1, 7]. In sub-Saharan Africa, adolescents aged 10–19 account for more than 90 % of global AIDS-related deaths within this age group and HIV incidence among adolescent girls and young women (AGYW) aged 15–24 in the region is the highest in the world [7]. In Zambia, in 2016, HIV prevalence among young women aged 20–24 was approximately 8.6 and 2.1 % among young men, and an estimated 60 % of adolescents aged 15–19 lacked comprehensive HIV knowledge [8, 9].

Meeting the SRH service needs of AYP requires increased investments in innovative strategies to improve the acceptability and accessibility of services [1]. To achieve this, meaningful engagement of AYP in the development of strategies to address their SRH needs is required [1–3, 10, 11]. Efforts to engage AYP and increase their access to services have previously been made in 21 Zambian and South African communities through the HPTN 071 (PopART) trial and the PopART for youth (P-ART-Y) sub-study [12, 13].

Between 2013 and 2017, the HPTN 071 (PopART) trial measured the impact of door-to-door delivery of a combination HIV prevention package, including universal testing and treatment, on HIV incidence at population level [14]. The aim of the nested P-ART-Y study was to determine the impact of the PopART intervention on HIV prevalence among adolescents aged 15–19 [13]. To communicate the studies, increase reach and engage the communities, adult and adolescent community advisory boards (CAB) were established in all study communities [15]. While both studies showed improvements in knowledge of HIV status and uptake of antiretroviral therapy, coverage was lower among AYP than in older age groups [12, 13]. Factors affecting HIV testing included perceived low risk of HIV, absence from households, refusal to participate in the study and challenges with obtaining parental consent for adolescents below 16 years [12, 13].

Building on lessons learnt through PopART, members of the study team co-developed, with AYP, an intervention to reach AYP in Zambia with comprehensive SRH. This process started with a consultation with adolescent community advisory board (aCAB) members in Lusaka and development of an initial intervention idea to deliver community-based, peer-led and incentivised SRH services. This paper describes the process of engaging AYP in finalising the design of the proposed comprehensive SRH intervention for AYP aged 15–24 in two peri-urban communities in Lusaka and shares lessons learnt in co-developing this intervention with AYP.

## Methods

This study was conducted between November 2018 and March 2019 in two high-density peri-urban communities in Lusaka, Zambia. The study formed part of the formative phase of a larger study to evaluate the impact of a co-designed SRH intervention on knowledge of HIV status and coverage of SRH services among AYP aged 15–24 [16]. The overall aim of this formative phase was to co-design the intervention with AYP. To achieve this aim, participatory qualitative research and discrete choice experiments (DCE) were used. This paper focuses on the qualitative component of the formative research study.

### Data collection

 We used participatory qualitative methods to assess AYP’s knowledge of HIV and SRH services (hereafter services), factors influencing AYP’s sexual behaviour and elicit AYP’s views on three core design elements of the proposed intervention: (1) the location of community-based spaces for service delivery, (2) type of individuals to deliver services, and (3) rewards to be provided through a proposed prevention points card (PPC) system (‘loyalty’ cards AYP could use to gain points and redeem them for rewards when accessing services).

**Fig. 1:**
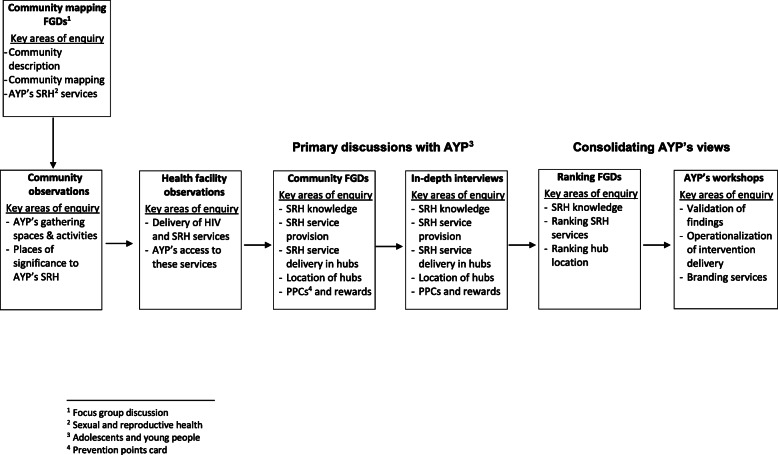
Sequence of data collection activities and key areas of enquiry per activity

Data were collected through focus group discussions (FGDs), in-depth interviews (IDIs) and observations. Embedded within FGDs and IDIs were participatory activities, including community mapping, concept mapping and ranking [17]. Activities were carried out to sequentially gather data and build on the enquiry (Fig. 1). As part of community entry, two community mapping FGDs were conducted in each community with adults (parents/guardians, community gate keepers, CAB and health committee members) and a mixed group of AYP aged 15–24. The community mapping FGDs established the context within which AYP’s SRH is situated through descriptions of the communities, livelihood options available, recreational activities and places of significance to AYP’s SRH.

Using community mapping data, we conducted four observations in each community through spiral walks [17, 18]. During spiral walks, we spoke to AYP about SRH (including service access), the activities they were engaged in and identified potential participants for subsequent research activities. In parallel, observations were carried out at the main government health facility in each community to observe the delivery of services to AYP.

Subsequently, ten FGDs and eight IDIs (collectively referred to hereafter as primary discussions) were conducted with AYP across the two communities to explore knowledge of services and views on the core components of the proposed intervention. To consolidate AYP’s views from the primary discussions, we conducted four ranking FGDs and two workshops with AYP in both communities. The ranking discussions aimed to identify the least and most preferred locations for service delivery and services to be offered through these locations. The workshops aimed to validate the research findings, gather AYP’s final views on how the proposed intervention would be operationalised (using role plays) and branding of services. Researchers took notes for all activities and during routine debriefing sessions conducted throughout the data collection period to discuss emerging issues [19]. Additionally, IDIs and FGDs were audio recorded.

### Participants

Study participants were purposively selected based on age, sex, where they lived and positions held in the study communities. In total, 215 AYP and 21 adults participated in the research. Among the AYP, six adolescent boys and young men (ABYM) and nine AGYW participated in both the primary and ranking FGDs. Their participation in both research phases facilitated continuity of discussions (building on previous findings), provided context to the issues being discussed and aided quick understanding for those participating for the first time. For similar reasons, some aCAB members and AYP who had participated in other data collection activities also attended the workshops. The socio-demographic characteristics of the study participants are presented in Table 1.

**Table 1:** Details of the research activities and the socio-demographics of the study participants

Activity	Number of activities	Participants	Age range (years)	Community one		Community two		Total
				No. of participants		No. of participants		
				Men/Boys	Women/Girls	Men/ Boys	Women/Girls	
Community mapping FGDs	2	Adults	25 – 75	7	4	3	7	21
	2	AYP^a^	16 – 24	4	4	6	5	19
Community groups FGDs^b^	2	Adolescent Boys	15 – 17	11	-	10	-	21
	2	Adolescent Girls	15 – 17	-	10	-	10	20
	2	Young men	18 – 24	5	-	10	-	15
	2	Young women	18 – 24	-	12	-	11	23
	2	aCAB^c^ members	18 – 24	2	7	3	6	18
Ranking FGDs	2	Adolescent boys and girls	15 – 17	3	5	5	5	18
	2	Young men and women	18 – 24	7	3	5	6	21
Workshops	2	Adolescent boys and girls and young men and women	15 – 24	15	17	19	16	67
In-depth interviews	2	Adolescent boys	15	1	-	1	-	2
	2	Adolescent girls	16 & 17	-	1	-	1	2
	2	Young men	23	1	-	1	-	2
	2	Young women	23 & 20	-	1	-	1	2
Total number of participants								251
Observations				Two health facility observations (one in each community).				
				Four community observations using the spiral walk approach (two in each community).				

^a^Adolescents and young people

^b^Focus group discussions

^c^Adolescent community advisory board

### Data analysis

Data were analysed thematically in two stages. Firstly, we conducted rapid analysis of the whole data set using matrix tables to quickly finalise the intervention design. Each data collection activity had a thematic matrix table, where responses to topic areas were completed soon after the activity. We used fieldwork notes written during data collection activities and debriefing sessions to complete the matrices. Data from the activity matrices were merged into a community specific matrix organised according to themes. The themes were pre-determined based on the topics of enquiry. Secondly, audio recordings were used to transcribe verbatim excerpts of discussions of particular interest and verify the accuracy of the completed activity matrices for finer analysis.

### Rigor

To ensure that our interpretations of the data collected were accurate, we triangulated information from different data collection methods and sources [19]. The workshops provided an opportunity for AYP to reaffirm the accuracy of our interpretation of the data [19].

### Ethics

 The study was approved by the London School of Hygiene and Tropical Medicine (ref: 15985) and the University of Zambia Biomedical Research Ethics committee (ref: 001-09-18). The Zambia national health research authority provided regulatory approval and the Ministry of Health permitted the study. All study procedures and methods were performed in accordance with scientific rigor and approved research guidelines and regulations by the ethics committees. For AYP aged < 18, parents/guardians provided written informed consent and adolescents provided written assent to participate. Participants aged ≥ 18 provided written informed consent. To uphold confidentiality, the two study communities are anonymised as communities 1 and 2.

## Results

The findings are presented according to the three phases of data collection, namely, community mapping, primary discussions and consolidation of AYP’s views.

### Community mapping: context of AYP’s SRH

The study communities had limited recreational facilities for AYP, and poverty and unemployment were reportedly high. AYP gathered in communal spaces such as bars, bus stops/stations, markets, sports facilities, schools and churches, to pass time or engage in piecework. Alcohol and drug abuse, peer pressure and lack of recreational facilities were factors identified as influencing AYP’s sexual behaviours. Some ABYM were involved in gangs which influenced decisions to access services and participation in crime, alcohol and drug use.

AYP had limited access to services, driven in part by a reluctance to access services from health facilities for fear of being reprimanded by health care workers (HCWs) and being seen accessing services considered culturally inappropriate for AYP by the communities. Additionally, not all services were readily available at the local health facilities. For example, voluntary medical male circumcision (VMMC) and cervical cancer screening services were not provided in community 2.

### Primary discussions

AYP, particularly ABYM, lacked knowledge of some services, including post and pre-exposure prophylaxis for HIV prevention and some contraceptive methods. Therefore, researchers had to inform AYP about services that were unclear to them. Discussions on most aspects of the core components of the proposed intervention yielded diverse views. Overall, AYP preferred delivery of most services in AYP specific spaces (hereafter hubs) in the community rather than at the health facility. However, for some services, including VMMC, cervical cancer screening and antenatal care, many AYP considered it important that these be offered at the health facility for reasons related to privacy and the level of expertise required to deliver these services.

*“In the community it is much better…they* [AYP] *say that mostly people are not comfortable coming to the clinic* [health facility]*…they are not comfortable with the workers* [HCWs]*…some say that they are afraid of meeting people they know,”* (young woman, 23 years, IDI community 1).

AYP suggested that hubs be located in common community gathering places, but the places they suggested were diverse, including markets, bus stops, churches, schools and sports fields. Although some AYP suggested that the hubs should be mobile and mounted in the suggested locations at different time periods, the majority preferred static hubs for ease of identification and access. Likewise, AYP had different opinions regarding who should provide services in the hubs. Some expressed preference for younger providers (peer support workers (PSWs)), while others considered the training (professional vs. lay) and sex of the providers more important. They further recommended that service providers should be welcoming, approachable and have a non-judgemental attitude towards AYP.

*“…sometimes you need fellow youths* [peers] *because we know that although they are testing people* [offering HIV testing], *they also experience what we experience…they know what they are talking about because they also go through the same things we experience,”* (Young man, 15–17 years FGD, community 2).

*“I think an adult who is a, who’s a health worker or kind of experienced, that would be better… for example my peer, we wouldn’t get along that much, but at least if I see that there is someone older, I’ll, I’ll try by all means to give that person respect and try to earn what I have gone there for…,”* (Young woman, 15–17 years FGD, community 1).

*“Sometimes it is difficult or embarrassing to open up to someone of the opposite sex,”* (20 year old young woman, IDI, community 2).

Distribution and use of PPCs was perceived as acceptable among all AYP, who readily understood the concept of points and rewards despite the concept of a “loyalty” card being new to them. They recommended community sensitisation prior to card distribution and made several suggestions regarding the appearance of the cards and information to be stored on them. A key suggestion was that the card should look attractive. Different groups and participants proposed different colours for the cards, with some suggesting different colours for each sex while others opposed this. The majority of AYP suggested an identification or serial number as a physical identifier to distinguish the cards while personal identifiers (address, name and photo), history of services accessed and points earned were to be encrypted on the card.

*“I think they [cards] can be the same and for sure there will just be different codes…because if you say that, me I have a green one and this one an orange one, that one, whatever colour. I will start asking myself, now I have a green one and that one has an orange one, maybe there is something wrong with my friend…,”* (Young man, aCAB FGD, community 1).

*“…the issue of details is essential, you have to take the details huh, but those details are not supposed to be written on the card. They are to be kept somewhere else and entered into the system…in a way that when you insert the card, it is registered by the number…but on the card there is no name…,”* (Young man, 15–17 years FGD, community 1).

AYP provided varying suggestions about how to allocate points for services accessed and redeeming rewards. For rewards, AYP unanimously agreed that costly rewards should be redeemed with more points. However, there was less agreement on the number of points to be allocated for services accessed. Some AYP thought that more points should be awarded to services considered psychologically harder for AYP to access, such as HIV testing, VMMC, cervical cancer and STI screening, while others thought that all services should be allocated the same number of points.

Commonly suggested rewards included: money, support with school requirements, airtime, phones, bags, caps, T-shirts and shoes. In fewer instances, AYP proposed tablets, laptops, wrist watches, employment and food supplies. Some rewards proposed by the study team, including movie tickets, Wi-Fi access and recognition of AYP as ambassadors of SRH services through appearing on a magazine cover or poster in the community, received varied responses, with some AYP either liking all, some or none of these suggestions. Similarly, there were diverging views regarding rewards promoting health (soap, toothpaste, mosquito nets). Those who liked these rewards suggested combining them as a package rather than as standalone rewards.

*“…on rewards, it’s just a suggestion, I was thinking that, if, maybe a person does not want to get a bag or T-shirt but maybe the person wants to keep the points for something bigger, I was thinking maybe even phones if many points have accumulated,”* (Young woman, aCAB FGD, community 2).

*“…it is not supposed to be fixed, such that you even know that every time when I come it is just soap I am going to get, soap always…today I get soap, next time toothpaste and toothbrush, next time a laptop bag, next time whatever. Better they are changing…,”* (Young man, 18–24 years FGD, community 2).

### Consolidating AYP’s views

Drawing on findings from the primary discussions, we presented the list of suggested locations for hubs and services to be offered through the hubs in ranking FGDs and asked AYP to rank them from the most to the least preferred. In both communities, AYP quickly identified locations that were close to each other and grouped them, suggesting that one hub could cater for everyone in these locations, and ranked their preferred locations. Factors influencing ranking included; population density, distance from other providers, availability of space and security to avoid vandalism.

*“I think the reason is ahh, coz that place has a lot of people* [highly populated] *and it is far away from health centres…so at least if you reach them, it will be, it will be good for them, they will enjoy these services,”* (Young man, Ranking FGD, 18–24 years, community 2).

In ranking services, and similar to the primary discussions, AYP had different views of what services should be offered in the hubs. Ranking was partly determined by their knowledge of the services. For instance, common services such as HIV testing were preferred compared to oral pre-exposure prophylaxis, which most AYP were unfamiliar with. Despite showing preferences for particular services and having diverse views of where services should be provided, AYP considered all services useful.

*“…you gave us to choose what services we do not want, in terms of all the services here, they are services whereby they are needed…,”* (Young man, Ranking FGD, 18–24 years, community 1).

**Fig. 2:**
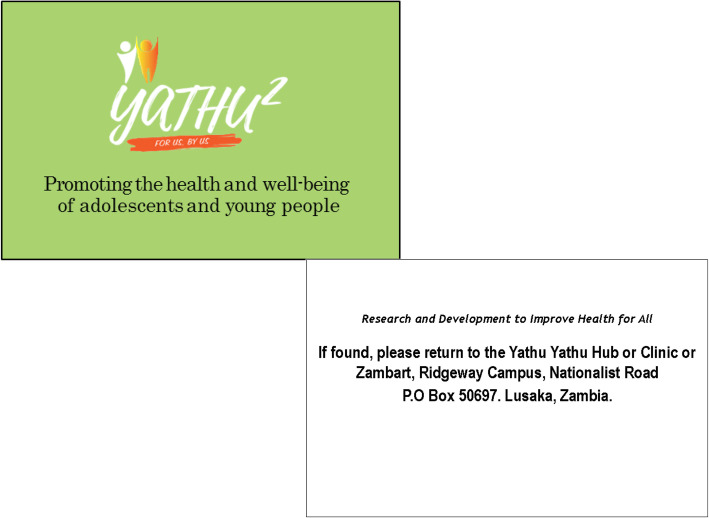
Final design of the prevention points card with the study logo

Consolidated research findings and the final intervention design were presented to AYP in workshops. Through these workshops, AYP provided further suggestions of how best to deliver the intervention. For example, they suggested having a PSW located at the health facility to facilitate referrals from the hubs. For branding, the majority of AYP chose lemon green, orange and white as colours to be used for the PPCs, hubs and other materials. Additionally, AYP provided input in the design of the study logo. Figure 2 shows the final design of the PPC with the study logo on it.

## Discussion

 This formative study used participatory qualitative methods to engage AYP in finalising the design of an SRH intervention for AYP aged 15–24. The study shows that AYP preferred delivery of most SRH services in static hubs located in common community gathering spaces with referrals to the health facility for services requiring more technical expertise. Preferences of service providers were varied, with AYP considering the age, sex and training when choosing their preferred type of provider. Use of PPCs was acceptable despite being a new concept and AYP suggested a wide range of rewards and considered it important that individuals choose rewards of their own preference.

Using participatory and human-centred approaches to inform the design or implementation of interventions has widely been recognised as important for tailoring interventions to meet end-user needs [17, 20–24]. Other qualitative studies that have engaged AYP to shape interventions have used similar approaches to those used in this study [25, 26]. Key strengths of our methods were the iterative process of engaging AYP and the use of a deliberative group discussion approach that enabled us to clarify topics that AYP were unfamiliar with so that they provide informed opinions about the intervention design [27]. Involvement of the same participants in different discussions aided AYP’s active participation and understanding of issues being discussed.

Our approach resonates with the increasing calls to involve AYP in programmes or research being conducted about them [1, 2, 10, 28]. Lack of involvement of adolescents in planning and implementing SRH services in public health facilities in Lusaka, Zambia, was identified as one of the factors that contributed to poor adherence to guidelines for providing adolescent-friendly services [29]. This study provides evidence of how to engage AYP in the design of an intervention that others can draw on.

Advocates of AYP engagement in research have argued that although AYP’s views may vary and it may be impossible to take equal consideration of the conflicting views, all opinions ought to be heard, respected and considered [30]. In our study, we also faced challenges with harmonising diverse views from AYP. The divergent views could partly be attributed to the wide age range of AYP we engaged, who were at different developmental stages with different SRH needs compared to other studies that involved narrower age ranges [25, 26]. However, the iterative process and triangulation of data from multiple sources enabled us to focus the findings and meaningfully inform the intervention design.

When designing this formative study, we anticipated gathering diverse views on some key components of the proposed intervention including, hub locations and service needs hence the ranking activity. While ranking worked well for hub locations, it did not work very well for services because of the number of proposed services and each service was distinct hence AYP considered all services to be essential. Lack of knowledge of some services could have also influenced how they were ranked. We recommend using pairwise ranking which can be used to compare each item with all the other items [31]. Overall consolidation of AYP’s views was also supported by the DCE [32].

Similar to our study findings, other studies have shown that AYP prefer to access services in community-based spaces compared to the health facility [33, 34]. A systematic review of interventions to prevent unintended and repeat pregnancy among young people in low and middle-income countries found that out of seven interventions that had a positive impact on contraceptive use, five were community-based [20]. On the contrary, other studies have argued that overcoming barriers of access to SRH services among AYP can be done by taking advantage of those who come to the health facility for other services such as postpartum and post abortion [20]. However, this would leave out those not accessing or uncomfortable with accessing services from the health facility as mentioned by AYP in our study.

The use of peers in delivering aspects of an AYP intervention has been recommended to build confidence and trust among young people hence focussing on addressing issues affecting them [30]. In this study, AYP recognised the critical role of PSWs in delivering services. A study in Zambia found that having peers as SRH service providers was important for delivering adolescent-friendly services [29]. As in our study, adolescents considered that PSWs were approachable and could easily relate to the SRH issues they experienced unlike older providers. Other studies have also shown the importance of friendly and approachable service providers in delivering adolescent friendly SRH services [35–37]. Similar to our study, adolescents in the other Zambian study preferred to access services from providers of the same sex as them [29].

Some studies have shown that incentives encourage AYP to access services and yield a positive impact on their SRH [38–41]. AYP in our study expressed interest in the PPCs. Some of the suggested rewards, including tablets, laptops, money and employment were unrealistic and offering such attractive incentives to promote SRH would be unethical. Other studies that have offered unconditional or conditional cash transfers have proved to be costly and unsustainable [20]. Therefore, careful consideration has to be given to the final intervention design with active participation of the AYP to ensure that the rewards system is responsive to their needs, enough to incentivise access to services yet sustainable to allow for scale-up.

## Limitations

The study explored the views of AYP aged 15–24. While this was the target group for the proposed intervention, the age range was wide. The SRH service needs of AYP are dependent on their stages of development hence the diverse views on most aspects of the core components of the proposed intervention. Although the study participants included school and non-school going AYP, a specific enquiry from AYP in school settings could have contributed to knowledge of suitable interventions to improve SRH among AYP in schools. Similarly, the study did not include non-binary AYP, therefore the process of engaging AYP described here might be different from that of engaging AYP of diverse gender, just as their preferences of how they would want services to be delivered would be different. Lastly, qualitative research study findings cannot be generalised to a larger population but can be applied in similar contexts [42]. Therefore, the study findings can only be applied to communities with similar characteristics as the study communities.

## Conclusions

Engaging AYP in designing an SRH intervention was feasible and supported the design of an intervention that is considered responsive to their needs. Although AYP’s suggestions were diverse and, at times, unrealistic or impractical, the process informed key components of the intervention. An iterative process of engaging AYP is recommended for developing a collaborative intervention design that is acceptable to AYP and feasible to implement.

## Data Availability

Analyses for this work were based on debriefing notes, matrices and listening to recordings to verify information in the matrices. Debriefing notes and recordings are not publicly available due to them containing information that could compromise research participant’s confidentiality. The matrices and data collection tools are available from the corresponding author on reasonable request.

## Abbreviations

ABYM: Adolescent boys and young men
ACAB: Adolescent community advisory board
AGYW: Adolescent girls and young women
AYP: Adolescents and young people
CAB: Community advisory boards
DCE: Discrete choice experiments
FGD: Focus group discussion
HCW: Health care workers
HIV: Human immunodeficiency virus
IDI: In-depth interviews
P-ART-Y: PopART for youth
PopART: Population effects of antiretroviral therapy to reduce HIV transmission
PPC: Prevention points cards
SRH: Sexual and reproductive health
STI: Sexually transmitted infections
VMMC: Voluntary medical male circumcision
